# Neutralization and clearance of GM-CSF by autoantibodies in pulmonary alveolar proteinosis

**DOI:** 10.1038/ncomms8375

**Published:** 2015-06-16

**Authors:** Luca Piccoli, Ilaria Campo, Chiara Silacci Fregni, Blanca Maria Fernandez Rodriguez, Andrea Minola, Federica Sallusto, Maurizio Luisetti, Davide Corti, Antonio Lanzavecchia

**Affiliations:** 1 Institute for Research in Biomedicine (IRB), Bellinzona 6500, Switzerland; 2 Institute of Microbiology, Eidgenössische Technische Hochschule (ETH), Zürich 8093, Switzerland; 3 Respiratory Disease Unit, IRCCS San Matteo Hospital Foundation, Pavia 27100, Italy; 4 Humabs Biomed SA, Bellinzona 6500, Switzerland

## Abstract

Pulmonary alveolar proteinosis (PAP) is a severe autoimmune disease caused by autoantibodies that neutralize GM-CSF resulting in impaired function of alveolar macrophages. In this study, we characterize 21 GM-CSF autoantibodies from PAP patients and find that somatic mutations critically determine their specificity for the self-antigen. Individual antibodies only partially neutralize GM-CSF activity using an *in vitro* bioassay, depending on the experimental conditions, while, when injected in mice together with human GM-CSF, they lead to the accumulation of a large pool of circulating GM-CSF that remains partially bioavailable. In contrast, a combination of three non-cross-competing antibodies completely neutralizes GM-CSF activity *in vitro* by sequestering the cytokine in high-molecular-weight complexes, and *in vivo* promotes the rapid degradation of GM-CSF-containing immune complexes in an Fc-dependent manner. Taken together, these findings provide a plausible explanation for the severe phenotype of PAP patients and for the safety of treatments based on single anti-GM-CSF monoclonal antibodies.

## Abstract (web summary)

Autoimmune pulmonary alveolar proteinosis is caused by autoantibodies to GM-CSF. Here the authors show that the individual autoantibodies only partially neutralize GM-CSF and that antibodies to at least three different epitopes are required to block GM-CSF bioavailability.

## Graphical abstract

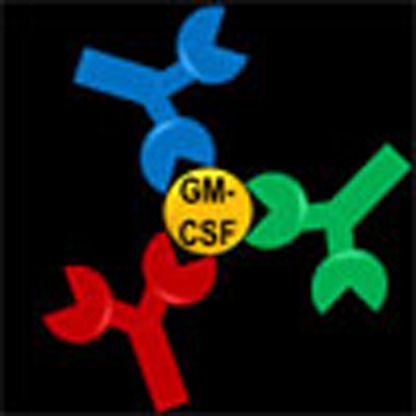

Autoantibodies against cytokines have been frequently reported both in healthy individuals and in patients with autoimmune or infectious diseases^1^. In several instances, a pathogenic role for cytokine autoantibodies has not been formally demonstrated, as it is the case for autoantibodies to interleukin (IL)-17 in patients with mucocutaneous candidiasis or autoantibodies to interferon (IFN)-γ in patients with mycobacterial infections^1^,^2^,^3^,^4^. In other instances, autoantibodies have been shown to cause severe pathology by neutralizing the biological activity of the target cytokine, as it is the case for autoantibodies to the granulocyte–macrophage colony-stimulating factor (GM-CSF) in autoimmune pulmonary alveolar proteinosis (PAP) and autoantibodies to erythropoietin in pure red-cell aplasia^5^,^6^. While in some cases autoantibody production has been linked to the administration of recombinant cytokines, such as erythropoietin, GM-CSF or IFN-β (refs 6, 7, 8), in most cases the stimuli that elicit the production of cytokine autoantibodies remain unknown.

The reason why cytokine autoantibodies may or may not cause pathology is not entirely clear. The prevailing view is that, when of enough affinity and present above a certain threshold of concentration, an autoantibody can neutralize the biological activity of the cytokine by simply binding and preventing its interaction with the cognate cellular receptor, a mechanism that can be recapitulated *in vitro* using cell proliferation bioassays with cytokine-dependent cell lines. Interestingly, however, several studies with toxins^9^,^10^,^11^ and cytokines^12^ demonstrated a synergy between different antibodies binding to the same molecule, suggesting that in some cases neutralization may be dependent on the production of antibodies targeting multiple antigenic sites, thus leading to the formation of immune complexes with the cytokine that can be efficiently cleared *in vivo*. In addition, it has been reported that monoclonal antibodies to IL-2, that neutralize the cytokine activity *in vitro*, can paradoxically enhance and extend IL-2 activity when injected *in vivo*^13^.

Autoimmune PAP is a rare and severe disease caused by the production of autoantibodies that neutralize the biological activity of GM-CSF. GM-CSF is essential for the terminal differentiation and function of alveolar macrophages that are responsible for the catabolism of surfactant lipids and proteins in the lungs^14^. The dysfunction of alveolar macrophages as a consequence of GM-CSF neutralization leads to the accumulation of lipoproteinaceous material within the alveoli, causing respiratory insufficiency and increased risk of infections^5^. In addition, GM-CSF deficiency results in impaired antimicrobial activity of neutrophils^15^. The pathogenic role of GM-CSF autoantibodies has been demonstrated by adoptive transfer of GM-CSF autoantibodies purified from PAP patients into non-human primates^16^. The sera of PAP patients contain levels of autoantibodies that exceed the pathogenic threshold of 10 μg ml^−1^, and that bind and neutralize GM-CSF at high titres^17^. A recent study reported the isolation of several monoclonal autoantibodies from PAP patients that showed different binding affinity and suggested that single human monoclonal antibodies neutralize the cytokine activity by blocking binding to the GM-CSF receptor^18^.

In this study we isolate a panel of monoclonal autoantibodies from PAP patients and show that single antibodies can only partially neutralize GM-CSF activity *in vitro*, depending on the experimental conditions of the bioassay, whereas three non-cross-competing antibodies completely neutralize GM-CSF activity. In addition, we show that, *in vivo*, single antibodies enhance the levels of bioavailable GM-CSF, while three non-cross-competing antibodies induce a rapid Fc-dependent clearance of the cytokine. Finally, we show that in most cases somatic mutations are critical for binding to GM-CSF, suggesting that clones that give rise to autoantibodies derive from memory rather than naive B cells.

## Results

### Characterization of GM-CSF autoantibodies from PAP patients

Peripheral blood mononuclear cells (PBMCs) and sera were collected from five chronic PAP patients of age ranging from 32 to 69 years. All patients had high serum titres of GM-CSF autoantibodies, ranging from 46 to 805 μg ml^−1^ (Supplementary Table 1). Memory B cells were isolated and immortalized with Epstein–Barr virus and CpG, as described^19^. The supernatants were screened for the presence of GM-CSF-specific IgG using enzyme-linked immunosorbent assay (ELISA) and 21 clones that produced GM-CSF autoantibodies (monoclonal antibodies, mAbs) were identified. The antibody V genes were sequenced and analysed using the IMGT (international ImMunoGeneTics information system) database^20^. Most antibodies were IgG1 (20/21) and used the κ light chain (19/21), with no clear preference for V gene usage. The load of somatic mutations was comparable to that characteristic of T-cell-dependent responses against non-self-antigens, ranging from 3.8 to 17.4% in the VH gene segment and from 0 to 14.5% in the VL gene segment. All antibodies were produced recombinantly as IgG1 and were tested for binding to GM-CSF (Table 1). The EC50 values determined using ELISA ranged from 16 to 836 ng ml^−1^ and the K_D_ values, as determined by surface plasmon resonance (SPR), ranged from 0.2 to 5.1 nM, consistent with high-affinity binding. Of notice, the kinetics values were heterogeneous. For instance, antibodies GCA7 and GCE402, which had comparable EC50 and K_D_ values, showed different kinetics, being GCA7 characterized by a low on-/low off-rate and GCE402 by a high on-/high off-rate. The properties of the monoclonal antibodies isolated are consistent with a polyclonal response of somatically mutated B cells.

### Binding to GM-CSF is dependent on somatic mutations

**Figure 1:**
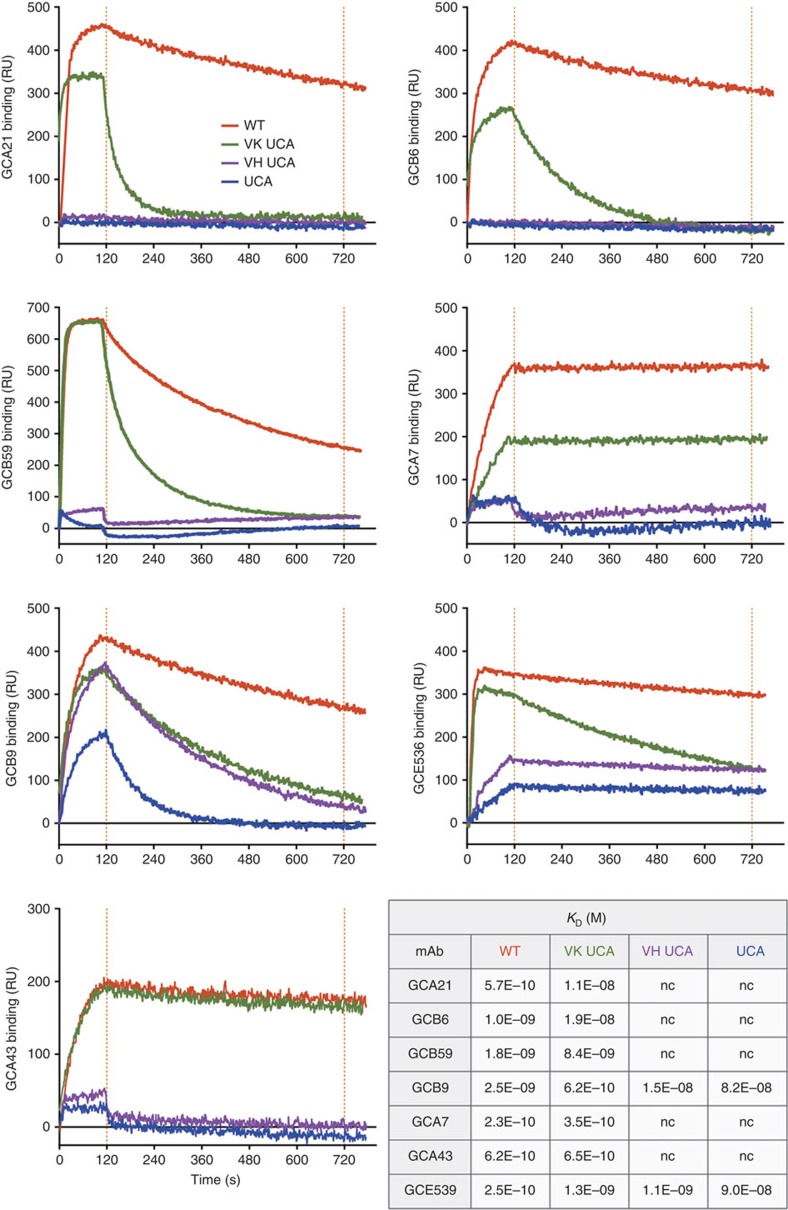
Somatic mutations critically contribute to the specificity of GM-CSF autoantibodies. Binding of WT (red), UCA (blue) and shuffled variants VK UCA (green) and VH UCA (violet) to GM-CSF as measured with SPR. The table shows equilibrium dissociation constants (*K*_D_).

The role of somatic mutations in the development of GM-CSF autoantibodies was investigated by testing versions of the autoantibodies in which the somatic mutations of the whole V(D)J gene were removed. We refer to these antibody variants as the unmutated common ancestors (UCA). We also produced shuffled variants in which only the VH or the VL were reverted to the UCA sequence. When analysed using SPR and ELISA, five out of the seven UCA antibodies tested did not bind to GM-CSF, while the remaining two showed a reduced, but still detectable, binding (Fig. 1 and Supplementary Table 2). Shuffling experiments showed that somatic mutations in the heavy chain played a major role in high-affinity binding. Since a precise identification of somatic mutations in the HCDR3 is not possible due to the difficulty of unequivocally assign the D segment and determine the junctional rearrangement, we also tested antibody variants in which only the V gene segment was reverted to the germline sequence, while the HCDR3 was left in the wild-type form. Interestingly, in four out of seven cases removal of mutations in the V segment was sufficient to abolish binding (Supplementary Table 2), providing formal evidence for the role of somatic mutations in determining autoantibody specificity.

### GM-CSF autoantibodies form high-molecular-weight complexes

**Figure 2:**
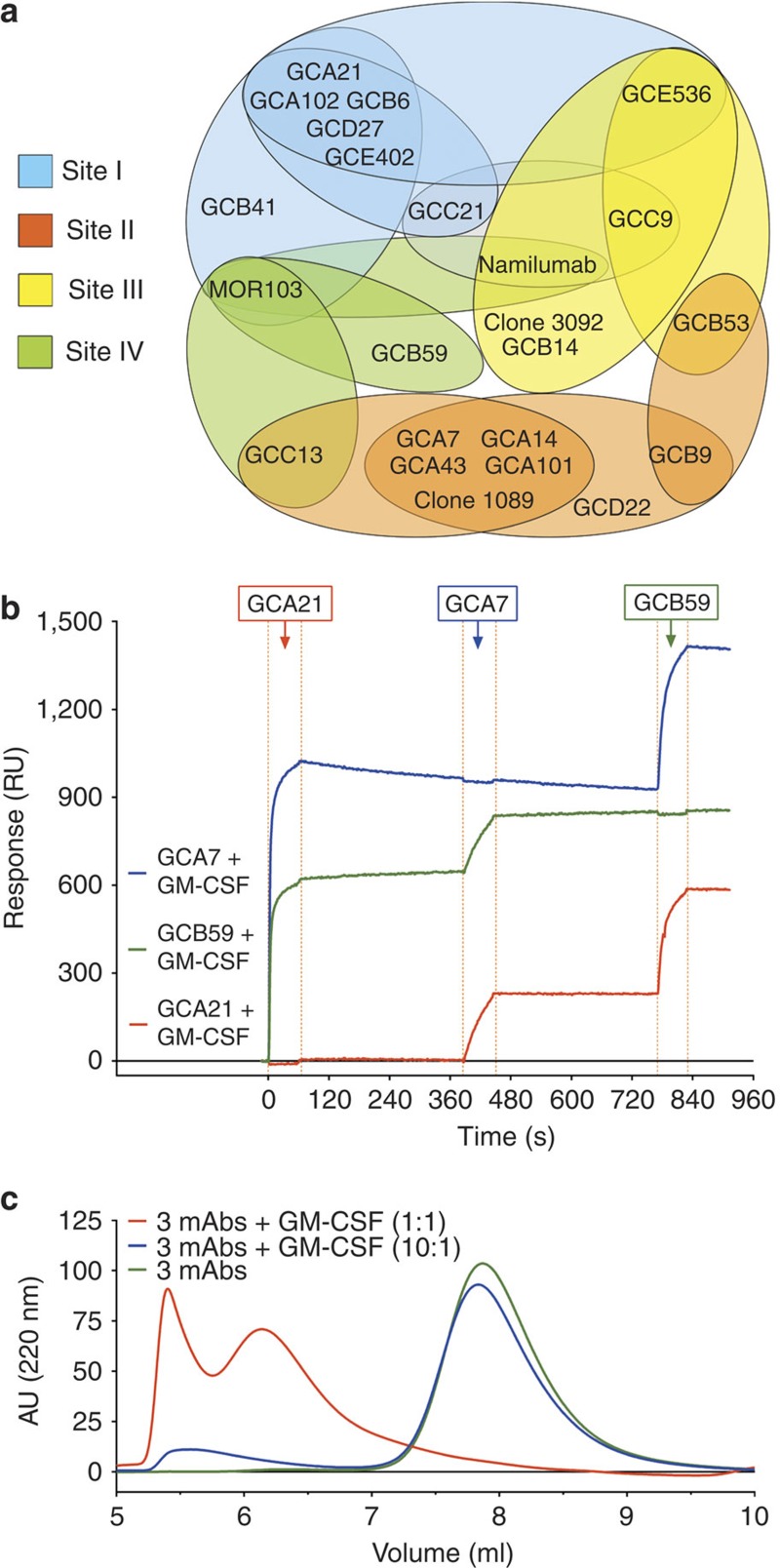
GM-CSF autoantibodies form high-molecular-weight complexes. (**a**) Map of the antigenic sites targeted by GM-CSF-specific autoantibodies as defined using SPR cross-competition. Four reference antibodies are in bold. (**b**) A multichannel chip coated with antibodies to site I (GCA21, red line), site II (GCA7, blue line) or site IV (GCB59, green line) was saturated with GM-CSF and serially exposed to an excess of the same antibodies. (**c**) The SEC-HPLC profile of samples containing the three non-cross-competing antibodies, alone or with GM-CSF added in equimolar concentrations (1:1) or in 10-fold antibody excess (10:1).

Using SPR cross-competition experiments we identified multiple antigenic sites on GM-CSF and a complete antigenic map based on a matrix of cross-competition experiments was generated (Fig. 2a). Sites I, II, III and IV are defined by four non-cross-competing autoantibodies GCA21, GCA7, GCB59 and GCC9. Interestingly, using SPR we could show that three non-cross-competing autoantibodies can bind simultaneously to a single molecule of GM-CSF (Fig. 2b). Furthermore, when GM-CSF was incubated with an excess of three antibodies, formation of high-molecular-weight immune complexes could be detected using size-exclusion chromatography-high-performance liquid chromatography (SEC-HPLC; Fig. 2c).

### Complete *in vitro* neutralization of GM-CSF by three antibodies

**Figure 3:**
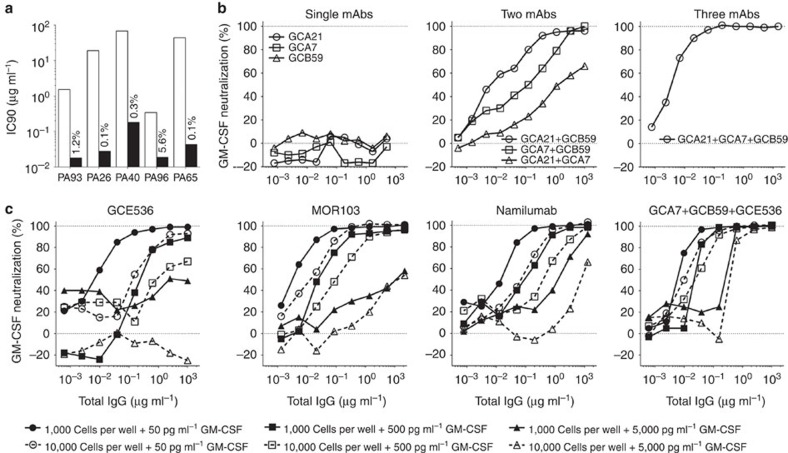
Potent *in vitro* neutralization of GM-CSF by a combination of three antibodies. A fixed amount of GM-CSF (final concentration 50 pg ml^−1^) was incubated with serial dilutions of one or more antibodies, added to TF-1 cells (10,000 per well), and cell proliferation was measured on day 3 by thymidine incorporation. (**a**) IC90 values of polyclonal IgG and affinity-purified polyclonal antibodies isolated from the serum of five PAP patients. The numbers indicate the percentage of anti-GM-CSF antibodies relative to total IgG. (**b**) Serial dilutions of single monoclonal antibodies or mixtures of two and three non-cross-competing antibodies were tested for their capacity to neutralize GM-CSF. (**c**) The sensitivity of the test was changed by varying the number of cells and the concentration of GM-CSF as indicated. Shown is for each experimental condition the inhibition obtained using single antibodies or a combination of three non-cross-competing antibodies.

The neutralizing activity of the autoantibodies was assessed by measuring their ability to inhibit the proliferation of TF-1 cells in response to recombinant GM-CSF. Polyclonal IgG and autoantibodies purified from the sera of PAP patients showed potent and complete neutralizing activity, with IC90 values ranging from 0.53 to 36 μg ml^−1^ and from 0.018 to 0.181 μg ml^−1^, respectively (Fig. 3a). From these values it was estimated that GM-CSF autoantibodies account for 0.1 up to 5.6% of total IgG in the serum of PAP patients (that is, 7.6 to 1,300 μg ml^−1^). These findings are consistent with previous reports^17^,^21^ and indicate that PAP patients have very high levels of GM-CSF autoantibodies capable of neutralizing the biologic activity of the cytokine.

Surprisingly, in the same bioassay, most monoclonal autoantibodies failed to neutralize GM-CSF (Fig. 3b). The only exception was GCE536 that neutralized GM-CSF activity with an IC90 value of 2.43 μg ml^−1^, while the therapeutic antibodies Namilumab and MOR103 (refs 22, 23) showed IC90 values of 0.80 and 0.16, respectively. Interestingly, when combined together, two non-cross-competing antibodies showed enhanced neutralizing activity both in terms of dose–response and percent inhibition, the combination of GCA21 (site I) and GCB59 (site IV) being the most effective (Fig. 3b). Furthermore, a combination of three non-cross-competing antibodies (GCA21, GCA7 and GCB59, specific for sites I, II and IV) led to a complete inhibition of proliferation with an IC90 value of 0.08 μg ml^−1^ (expressed as the total concentration of the three mAbs), which was lower than that of the therapeutic antibodies MOR103 and Namilumab. A strong synergy between three non-cross-competing antibodies was observed for most combinations tested, with IC90 values comparable to those of affinity-purified antibodies from PAP sera (Supplementary Fig. 1).

Considering the law of mass action, we hypothesized that in the presence of a single antibody a small fraction of GM-CSF may continuously dissociate from the antibody and become available to trigger the high-affinity GM-CSF receptor. In contrast, in the presence of three antibodies, GM-CSF may be sequestered irreversibly in stable immune complexes. As expected from the law of mass action, we found that by varying the cell number and the GM-CSF concentration the sensitivity of the assay was dramatically affected. In particular, lowering the number of TF-1 cells and the concentration of GM-CSF led to a more sensitive test that showed increased neutralization by single and multiple antibodies (Fig. 3c). In contrast, when high number of TF-1 cells and high doses of GM-CSF were used, even the most potent neutralizing antibodies MOR103 and Namilumab, failed to neutralize GM-CSF, even when present in a 400-fold molar excess. Strikingly, in all conditions, a combination of three non-cross-competing antibodies was capable of completely neutralizing GM-CSF.

### Fc-dependent clearance of GM-CSF immune complexes *in vivo*

**Figure 4:**
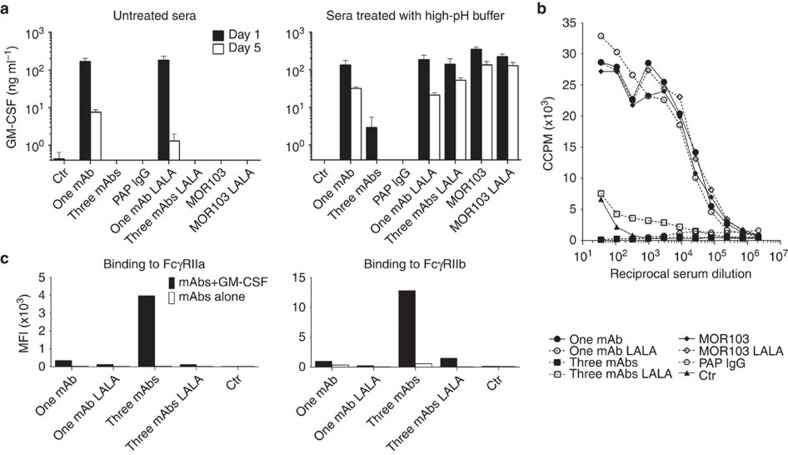
Fc-dependent clearance of GM-CSF immune complexes *in vivo*. (**a**) Female Balb/c mice (five per group) were injected with 100 μg of mAb, either GCA21 (one mAb) or GCA21+GCA7+GCB59 (three mAbs) in the IgG or IgG-LALA format, or with 2 mg total IgG from a PAP patient, followed by 2 μg GM-CSF after 16 h. Sera were collected after 1 or 5 days, and GM-CSF concentrations were measured using ELISA in untreated serum and in serum treated at pH 11.6 to dissociate immune complexes. Shown is the GM-CSF concentration on day 1 and on day 5 in untreated serum (left) or alkaline-treated serum (right);error bars indicate s.d. (**b**) Proliferation of TF-1 cells in response to different dilutions of serum of mice injected 24 h before with GM-CSF and the indicated antibodies. (**c**) Binding of GM-CSF immune complexes formed by one or three antibodies (in the IgG1 or IgG1-LALA format) to TZM-bl cells expressing FcγRIIa or FcγRIIb, as measured with flow cytometry using an anti-IgG Fc-specific antibody.

Having established that GM-CSF can form complexes with three antibodies resulting in efficient *in vitro* neutralization of the cytokine biological activity, we were interested in understanding the effect of single versus multiple autoantibodies *in vivo*. Mice were injected with a total of 100 μg of single or multiple monoclonal antibodies or with 2 mg of the IgG fraction isolated from the serum of a PAP patient, followed by injection of 2 μg of human GM-CSF. At different time points serum was collected and the amount of GM-CSF present was measured by a sandwich ELISA using an antibody specific for site II for capture and site I for detection. The assay was performed on serum either untreated or after alkaline treatment to dissociate the immune complexes (Supplementary Fig. 2). In the absence of antibodies, the injected GM-CSF disappeared rapidly from the serum and was undetectable after 24 h (Fig. 4a). In contrast, when single antibodies (GCA21 or MOR103) were used, high levels of GM-CSF were recovered from serum on day 1 and were still present on day 5. Of note, GM-CSF detection required alkaline dissociation in the case of MOR103 but not for GCA21, consistent with the different dissociation rates of the two antibodies (Table 1). In striking contrast, when mice received three non-cross-competing antibodies (GCA21, GCA7 and GCB59) or PAP IgG, GM-CSF was rapidly cleared since only low or undetectable amounts of the cytokine could be detected in the day-1 and day-5 sera, respectively, after alkaline dissociation.

To address the possible role of Fc receptors in the clearance of GM-CSF, we tested the same antibodies in a variant form, called LALA, which does not bind to C1q or to Fcγ receptors. Similarly to the wild-type antibodies, single LALA antibodies led to an increase in GM-CSF levels in the serum. However, in contrast to what observed for three wild-type antibodies, three LALA antibodies failed to clear GM-CSF, which was quantitatively recovered in the sera following alkaline dissociation even on day 5 (Fig. 4a).

To ask whether the antibody-bound GM-CSF would be bioavailable, we tested the sera of mice for their ability to support TF-1 proliferation (Fig. 4b). Sera of mice receiving GCA21 or MOR103 led to a robust proliferation of TF-1 cells, consistent with a GM-CSF dissociation rate sufficient to engage the cytokine receptor. In contrast, sera of mice receiving three wild-type antibodies or PAP IgG were not able to stimulate proliferation, consistent with clearance of the immune complexes *in vivo*. In addition, although containing high level of GM-CSF, sera of mice receiving three LALA antibodies were not stimulatory, a finding consistent with irreversible sequestration of GM-CSF in stable immune complexes.

To further address the role of Fcγ receptors, we tested immune complexes formed between GM-CSF and wild-type or LALA antibodies for their capacity to bind to TZM-bl cells expressing different Fcγ receptors. Strong binding was observed only on FcγRIIa- and FcγRIIb-expressing cells and when immune complexes were formed by three wild-type, but not LALA, antibodies (Fig. 4c and Supplementary Fig. 3). Taken together, the above results indicate that single antibodies, even when potently neutralizing *in vitro*, increase the half-life of GM-CSF and build up a circulating pool of bioavailable cytokine. In contrast, three or more antibodies lead to the formation of immune complexes that are efficiently cleared through an Fc-dependent mechanism.

### Low levels of GM-CSF autoantibodies in healthy donors

**Figure 5:**
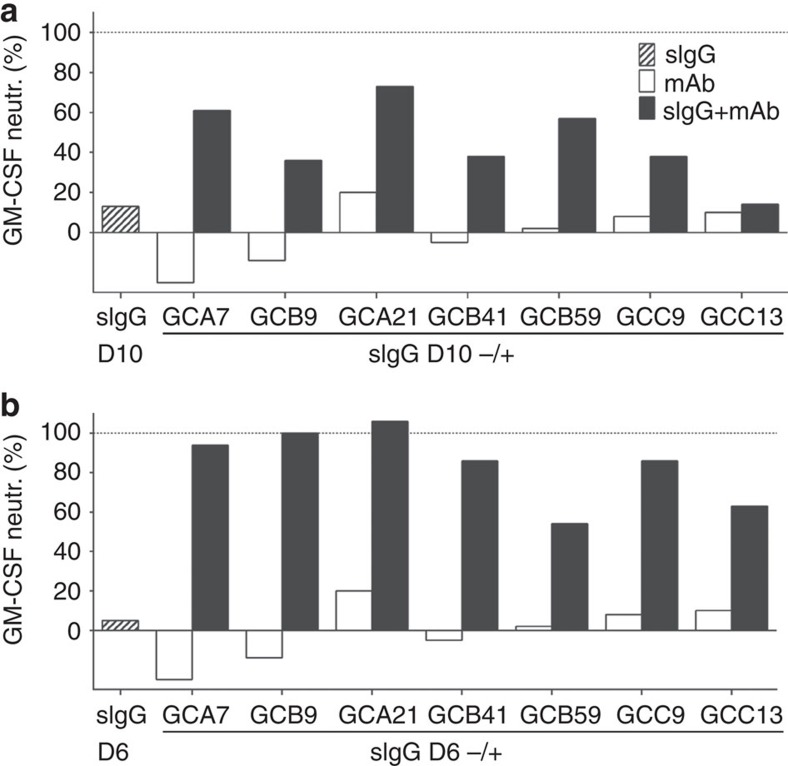
Detection of low levels of GM-CSF autoantibodies in healthy donors using a complementation assay. (**a,b**) IgG purified from the serum of two healthy donors (D10 and D6) were tested in the presence or absence of single monoclonal antibodies for their capacity to neutralize GM-CSF using the TF-1 bioassay (50 pg ml^−1^ GM-CSF and 10,000 cells per well).

To address whether low levels of autoantibodies to GM-CSF may be found in the serum of healthy donors, we took advantage of the synergy between autoantibodies in GM-CSF neutralization. When tested for their capacity to neutralize GM-CSF bioactivity on TF-1 cells, the IgG fraction isolated from serum of healthy donors did not show detectable neutralizing activity. However, when serum IgG was supplemented with a single non-neutralizing monoclonal antibody, a clear neutralizing activity was measured, which varied depending on the serum donor and on the site specificity of the antibodies used. In one case we could observe complete neutralization using the serum IgG from donor 6 (D6) combined with GCB9 and GCA21 antibodies (Fig. 5). These results suggest that low levels of anti-GM-CSF antibodies devoid of neutralizing activity are present in the sera of healthy donors and can be detected using a sensitive complementation assay.

## Discussion

Our study identifies two mechanisms by which polyclonal anti-GM-CSF antibodies produced by PAP patients inhibit the biological activity of GM-CSF. The first is the irreversible sequestration of GM-CSF in high-molecular-weight immune complexes that can be readily detected *in vitro* using cell proliferation bioassays. The second is the *in vivo* Fc-dependent degradation of immune complexes formed between GM-CSF and multiple autoantibodies.

The autoantibodies isolated show affinities in the nanomolar range and distinct kinetics of binding, differing in particular in their off-rates. Overall, they were comparable to those reported in a recent study^18^. Interestingly, while this study claimed that all the antibodies were capable of neutralizing GM-CSF in the TF-1 bioassay, we show that neutralization by single antibodies is strictly dependent on the concentration of the cytokine and the number of receptors in the system, a finding that can be explained by the reversibility of antibody–antigen interaction, according to the law of mass action, and the affinity of GM-CSF receptor for its ligand, which is higher than that of antibodies^24^,^25^. It was therefore interesting to discover that, when added together, three antibodies that bind to nonoverlapping sites can completely neutralize GM-CSF bioactivity, irrespective of the concentration of cytokine or receptor. When tested under stringent conditions, a cocktail of three antibodies was, on a weight basis, more potent than two anti-GM-CSF antibodies that are currently developed for therapy of autoimmune and inflammatory diseases^22^,^23^,^26^. We envisage that, when complexed with three antibodies, GM-CSF becomes completely sequestered and no longer available for interaction with the receptor.

A key observation in this study was that, when injected in mice, single antibodies led to the accumulation of a large pool of long-lived GM-CSF that was still able to dissociate and trigger the receptor, as shown by the capacity of the sera to stimulate proliferation of TF-1 cells. The capacity of a single antibody to build up a large cytokine reservoir, as we observed for GM-CSF, could be the basis of the enhancing activity of monoclonal antibodies to common gamma-chain cytokines *in vivo*^13^. In striking contrast, we showed that three antibodies formed immune complexes that were rapidly degraded *in vivo* in an Fc-dependent manner. The degradation of GM-CSF induced by polyclonal antibodies is reminiscent of a previous study where three antibodies were shown to cause degradation of IL-6 *in vivo*^12^. Our result extend this concept to autoantibodies and suggest that degradation is the main mechanism by which polyclonal autoantibodies, such as those found in the serum of PAP patients, can lead to complete clearance of the cytokine, thus explaining the severe phenotype characteristic of these patients. In summary, our results suggest a two-step mechanism by which polyclonal antibodies lead to a severe GM-CSF deficiency: first, they irreversibly sequester GM-CSF thus completely preventing its interaction with the receptor and, second, by inducing its degradation in an Fc-dependent manner. Our findings also help explain why PAP has not been reported following administration of an anti-GM-CSF monoclonal antibody in humans^26^. We suggest that the main effect of the therapeutic anti-GM-CSF antibody would be to redistribute the cytokine into the circulation, thus decreasing its concentration in inflammatory sites, while leaving enough free GM-CSF for the development of alveolar macrophages. In addition, it is worth considering that systemically administered IgG accumulates preferentially in inflamed tissues as compared with the lung^27^,^28^.

An interesting observation of this study is related to the role of somatic mutations and the presence of low levels of GM-CSF antibodies in healthy individuals. The finding that the UCA of the autoantibodies did not bind, or showed minimal binding to GM-CSF, is consistent with previous studies on the role of somatic mutations in determining the specificity of autoantibodies to desmogleins or DNA in patients with pemphigus or lupus^29^,^30^,^31^. In contrast, we observed that the UCA versions of antiviral antibodies showed only a slightly reduced binding to the specific antigen^32^,^33^,^34^. These findings support the notion that autoantibodies are preferentially generated from activated memory B cells that have been triggered and have undergone somatic mutation in response to foreign antigens. It is tempting to speculate that the preferential origin of autoantibodies from memory B cells is because of the fact that these cells have a lower activation threshold or may be more difficult to anergize as compared with naive B cells. In addition, it has been suggested that memory B cells may be able to efficiently prime naive T cells^35^.

GM-CSF autoantibodies have been described not only in PAP patients but also in the serum of healthy donors or in patients with inflammatory bowel disease with normal pulmonary function^21^,^36^. Building our findings, we developed a sensitive complementation assay that detects in the serum of healthy donors non-neutralizing GM-CSF autoantibodies based on their capacity to synergize with non-neutralizing monoclonal autoantibodies. These findings suggest that healthy donors may have anti-GM-CSF antibodies in serum at low concentrations and/or in combinations that cannot promote GM-CSF sequestration and degradation. Conversely, PAP patients developed high levels of GM-CSF autoantibodies to multiple sites that form immune complexes that mediate sequestration and degradation of the cytokine.

## Methods

### Isolation and production of monoclonal antibodies from PAP patients

Peripheral blood samples were obtained from five PAP patients with high levels of GM-CSF autoantibodies. Determination of the serum level of GM-CSF autoantibodies was performed in the Laboratory of the Rare Lung Disease Consortium at the Cincinnati Children’s Hospital Medical Center, as described^17^,^37^. Briefly, ELISA plates (MaxiSorp, Nunc) were coated with 1 μg ml^−1^ of recombinant human GM-CSF (Miltenyi Biotec), blocked with blocking solution Stabilcoat (Surmodics, Eden Prairie, MN, USA) and incubated with titrated sera (1/101, 1/3,001, 1/6,001 and 1/12,001), followed by incubation with horseradish peroxidase-conjugated anti-human IgG F(ab′)2 fragment. Plates were then washed, substrate (tetramethylbenzidine; T4444, Sigma-Aldrich) was added followed by 1 N H_2_SO_4_ and the plates were read at 450 nm. Serum concentration of GM-CSF autoantibodies was determined from a GM-CSF-autoantibody polyclonal reference standard by quadratic regression analysis using Microsoft Excel (Microsoft Corp, Seattle, WA, USA). The Disease Severity Score, a combination of presence and absence of symptoms and degree of PaO2, was determined according to the classification previously described^38^. Memory B cells were isolated from cryopreserved or fresh PBMCs using anti-fluorescein isothiocyanate (FITC) microbeads (Miltenyi Biotec) following staining of PBMCs with CD22-FITC (BD Phamingen), and were immortalized with Epstein–Barr virus and CpG in multiple wells as described previously^19^. Culture supernatants were tested for binding to recombinant human GM-CSF (Gentaur, 04-RHUGM-CSF) using ELISA. cDNA was synthesized from positive cultures and both heavy chain and light chain variable regions were sequenced. All monoclonal antibodies were produced recombinantly as IgG1 by transient transfection of HEK 293 Freestyle Cells (Invitrogen) using polyethylenimine, and tested for binding to GM-CSF using ELISA. The study was conducted in accordance with the Declaration of Helsinki guidelines and was approved by the Ethics Committee of IRCCS San Matteo Hospital Foundation of Pavia, Italy. All patients gave informed consent.

### Sequence analysis of antibodies and reversion to germline

The usage of VH and VL genes and the amount of somatic mutations were determined by analysing the homology of VH and VL sequences of mAbs to known human V, D and J genes by the IMGT database^20^. Sequences of UCA were determined by reverting mutations to the germline sequence while retaining the original CDR3 junctions and terminal deoxy-nucleotidyl transferase N nucleotides. UCA sequences in which putative mutations of the HCDR3 were not removed were also determined. VH UCA, VH UCA (HCDR3 WT) and VL UCA nucleotide sequences were synthesized by Genscript, and their accuracies were confirmed by sequencing. Four different versions of seven selected mAbs were produced recombinantly: UCA, UCA–HCDR3 WT, and shuffled mAbs either with VH UCA+wild-type VL or with wild-type VH+VL UCA.

### Antibody purification and ELISA assays

Human mAbs and total IgG from PAP sera were purified by protein A or protein G chromatography (GE Healthcare). Total IgG from healthy donors were purified using the HiTrap Protein A HP columns (GE Healthcare) and concentrated using Amicon Ultra filter units (100 K, Millipore). Total GM-CSF antibodies were affinity-purified from PAP sera using magnetic beads (Invitrogen) conjugated with human GM-CSF. Total IgGs were quantified using ELISA plates coated with anti-human IgG (SouthernBiotech) using Certified Reference Material 470 (ERMs-DA470, Sigma-Aldrich) as standard. Binding to GM-CSF was tested by ELISA using 384-well SpectraPlates (PerkinElmer) for primary screenings or 96-well MaxiSorp plates (Nunc) for any following test. Briefly, ELISA plates were coated with 1 μg ml^−1^ of recombinant human GM-CSF (Gentaur), blocked with 1% BSA and incubated with titrated antibodies, followed by AP-conjugated anti-human IgG secondary antibodies (SouthernBiotech). Plates were then washed, substrate (p-NPP, Sigma) was added and plates were read at 405 nm. EC50 (ng ml^−1^) was calculated for every sample by nonlinear regression analysis using the GraphPad Prism 5 software.

### SPR assays

Protein A (450 nM) was stabilized in 10 mM acetate buffer, pH 4.5, and immobilized on a EDC/NHS pre-activated ProteOn sensor chip (Bio-Rad) through amine coupling; unreacted groups were blocked by injection of ethanolamine HCl (1 M). HEPES-buffered saline (HBS; 10 mM HEPES, pH 7.4, 150 mM NaCl, 3 mM EDTA, 0.005% surfactant Tween-20) was used as running buffer. All injections were made at the flow rate of 100 μl min^−1^. Monoclonal antibodies were diluted in HBS (200 nM) and injected on the protein A-coated chip for capturing, followed by injection of different concentrations of human GM-CSF (400, 200, 100, 50 and 25 nM); one channel of the chip was injected with HBS and used as reference for the analysis. Injection time and dissociation time were 120 and 600 s, respectively. Each binding interaction of mAbs with GM-CSF was assessed using a ProteON XPR36 instrument (Bio-Rad) and data processed with the ProteOn Manager Software. *K*_a_, *K*_d_ and *K*_D_ were calculated by applying the Langmuir fit model. To determine the epitope specificity, GM-CSF autoantibodies (150 mM) were directly immobilized on a sensor chip, followed by injection of GM-CSF (100 nM) and autoantibodies (200 nM). To assess simultaneous binding to GM-CSF, different mAbs (200 nM each) were serially injected after the GM-CSF capture (50 nM). Injection time and dissociation time were 60 and 20 s, respectively.

### Size-exclusion HPLC

Three non-cross-competing mAbs were diluted in PBS singularly or as a three-antibody-combination (10 μg of total antibody amount) and were mixed with GM-CSF (1:1 or 10:1 molar ratios) for 1 h at room temperature (RT). Samples were analysed with the Agilent 1100 HPLC machine using TSK-GEL G3000SW columns (Tosoh, bed volume: 13 ml, void volume: 4.6 ml) with PBS as the mobile phase (flow rate: 1 ml min^−1^). A universal solvent 2-μm filter (Agilent) was put between injector and column. Detection was performed using a Variable Wavelength Detector (Agilent) with ultraviolet absorption at 220 nm.

### TF-1 proliferation bioassays

TF-1 cells (Cell Lines Service) were maintained in RPMI 1640 medium supplemented with 10% fetal bovine serum (Hyclone), 1% GlutaMAX, 1% Penicillin/Streptavidin, 1% non-essential amino acids, 1% sodium pyruvate, 1‰ 2-mercaptoethanol (all from GIBCO), 5 ng ml^−1^ human GM-CSF (Gentaur) and 10 ng ml^−1^ human IL-3 (ImmunoTools). Cells were grown at 37 °C in a humidified incubator with 5% CO_2_. A GM-CSF neutralization assay was performed by serially diluting mAbs (or a combination of mAbs, total IgG or affinity-purified antibodies) in growth medium with neither GM-CSF nor IL-3, adding GM-CSF at a concentration of 100 pg ml^−1^, and preincubating in 96-well flat-bottom cell culture plates (Costar) at 37 °C for 1 h. TF-1 cells were washed five times, diluted in the growth medium with neither GM-CSF nor IL-3, and 10,000 cells per well were seeded (final GM-CSF concentration equal to 50 pg ml^−1^). In other tests, GM-CSF was used at the final concentration of 500 and 5,000 pg ml^−1^, and 1,000 cells per well were seeded. Cells with or without GM-CSF in absence of antibodies were used as control to determine maximum and minimum levels of cell proliferation. Plates were incubated at 37 °C in a humidified incubator with 5% CO_2_ for 72 h, and cell proliferation was measured after 6-h incubation with 0.2 μCi per well of [3H]-thymidine (PerkinElmer). GM-CSF neutralization was calculated as percentage of inhibition of TF-1 growth with the following formula: [1−(CCPM of a single well−average CCPM of control cells grown without GM-CSF) × (average CCPM of control cells grown with GM-CSF−average CCPM of control cells grown without GM-CSF)^−1^] × 100 (CCPM=corrected counts per minute). IC90 (μg ml^−1^) was calculated for every sample by a nonlinear regression analysis using the GraphPad Prism 5 software. In some experiments mouse sera were titrated in TF-1 growth medium and preincubated at 37 °C for 30 min. TF-1 cells were washed and seeded (1,000 cells per well). A titration of GM-CSF (60,000–0.3 ng ml^−1^) was added as growth control. CCPM of each single well were plotted against the serum titration.

### *In vivo* clearance of GM-CSF immune complexes

BALB/C mice were supplied by The Jackson Laboratory, USA; Harlan, Italy; Charles River, Germany, Italy. Animal experiments were approved by the Cantonal Autorithies of Cantone Ticino. Groups of 6- to 8-week-old female BALB/c mice were injected intravenously with 100 μg of purified mAbs or 2 mg of total IgG purified from PA96 patient. After 16 h, 2 μg of human GM-CSF were injected. Serum samples were collected on day 1 and day 5. GM-CSF was quantified with a sandwich ELISA. Briefly, 10 μg ml^−1^ of an antibody that bound to site II of GM-CSF was used to coat 96-well Maxisorp plates (Nunc), which were then blocked with PBS+10% FBS (Gibco). All sera and GM-CSF, which was used as standard (range 3.4–600,000 pg ml^−1^), were titrated and tested in parallel under different conditions: in one plate all samples were supplemented with 25% (vol/vol) of an alkaline dissociation buffer (2.5% Triton X-100, 2 M ethanolamine, 0.15 M NaCl, pH 11.6), in the other plate all samples were supplemented with 25% (vol/vol) of PBS+10% FBS. Plates were left overnight at RT. Detection of captured GM-CSF was made with 1 μg ml^−1^ of a biotinylated antibody that bound to site I of GM-CSF for 1 h at RT, followed by binding of 0.5 μg ml^−1^ streptavidin-AP (Jackson ImmunoResearch) for 1 h at RT. Plates were then washed, substrate (p-NPP, Sigma) was added and plates were read at 405 nm.

### Binding of GM-CSF immune complexes to FcγR

Four TZM-bl cell lines (NIH AIDS Research & Reference Reagent Program) each transfected with a specific Fcγ receptor (FcγRI, FcγRIIa, FcγRIIb or FcγRIIIa) were maintained in DMEM medium supplemented with 10% fetal bovine serum (Hyclone), 0.025 M Hepes, 10 μg ml^−1^ Gentamicin and 20 μg ml^−1^ Blasticidin. Untransfected TZM-bl cells were used as negative control and were maintained in DMEM medium supplemented with 10% fetal bovine serum (Hyclone) and 2% Penicillin/Streptavidin. Cells were grown at 37 °C in a humidified incubator with 5% CO_2_. Expression of specific FcγRs was assessed by staining TZM-bl cells with FITC-conjugated anti-CD64 (anti-FcγRI), anti-CD32 (anti-FcγRIIa and anti-FcγRIIb) and anti-CD16 (anti-FcγRIIIa) antibodies (all from BD Pharmingen). Untransfected and transfected TZM-bl cells were washed with staining buffer (PBS with 10% fetal bovine serum and 2 mM EDTA) and seeded in 96-well plates at a density of 50,000 cells per well. A single anti-GM-CSF mAb (GCA21) or a combination of three non-cross-competing mAbs (GCA21, GCA7 and GCB59) at a final concentration of 2.5 μg ml^−1^ were mixed with 0.05 μg ml^−1^ GM-CSF, or staining buffer (PBS with 10% fetal bovine serum and 2 mM EDTA). The LALA versions of all antibodies and a mAb with a different specificity were included as controls. Samples were incubated at 37 °C for 30 min to allow the formation of immune complexes and then cooled down to 4 °C before adding them to TZM-bl cells for 30 min. Cells were washed twice and stained with anti-human IgG Fcγ fragment specific F(ab′)_2_ fragment (Jackson ImmunoResearch). Samples were analysed on BD FACSCanto (BD Biosciences) and the median intensity fluorescence was analysed and compared between samples.

## Additional information

**How to cite this article:** Piccoli, L. *et al.* Neutralization and clearance of GM-CSF by autoantibodies in pulmonary alveolar proteinosis. *Nat. Commun.* 6:7375 doi: 10.1038/ncomms8375 (2015).

## Supplementary Material

Supplementary Information Supplementary Figures 1-3, Supplementary Tables 1-2

## Figures and Tables

**Table 1:** Binding properties and V gene usage of GM-CSF autoantibodies isolated from patients with pulmonary alveolar proteinosis.

**mAb**	**Patient**	**Isotype**	**EC50 (ng ml** ^**−1**^ **)**	***K***_**a**_ **(M s**^**−1**^**)**	***K***_**d**_ **(s**^**−1**^)	***K***_**D**_ **(M)**	**Heavy chain VDJ genes (% identity to GL**)			**Light chain VJ genes (% identity to GL)**	
GCA7	PA93	IgG1 (κ)	186.8	2.4E+05	6.0E−05	3.8E−10	*VH3-66* (91.2)	*D3-10*	*JH4* (93.8)	*VK4-1* (96)	*JK3* (100)
GCA14	PA93	IgG1 (κ)	213.0	3.1E+05	9.6E−05	3.1E−10	*VH1-58* (92.4)	*D3-9*	*JH6* (84.9)	*VK4-1* (97.6)	*JK2* (86.8)
GCA21	PA93	IgG1 (κ)	59.4	9.5E+05	6.5E−04	6.9E−10	*VH3-30-3* (83.3)	*D2-15*	*JH2* (84.9)	*VK1-5* (92.8)	*JK4* (97.2)
GCA43	PA93	IgG1 (κ)	835.6	1.7E+05	1.6E−04	9.8E−10	*VH1-69* (91.7)	*D5-24*	*JH3* (88)	*VK4-1* (95.3)	*JK2* (100)
GCA101	PA93	IgG1 (κ)	291.5	3.7E+05	1.1E−04	3.9E−10	*VH4-59* (90.5)	*D1-26*	*JH2* (94.3)	*VK4-1* (91.6)	*JK2* (92.1)
GCA102	PA93	IgG1 (κ)	208.1	3.8E+05	2.8E−04	7.8E−10	*VH3-23* (90.7)	*D6-13*	*JH6* (90.3)	*VK1-16* (94.3)	*JK4* (97.4)
GCB6	PA26	IgG1 (κ)	92.4	4.9E+05	2.8E−04	5.6E−10	*VH3-23* (87.5)	*D1-7*	*JH 6* (80.7)	*VK1-16* (95.7)	*JK4* (88.9)
GCB9	PA26	IgG1 (κ)	228.3	2.1E+05	7.6E−04	3.6E−09	*VH1-18* (82.6)	*D4-23*	*JH4* (85.4)	*VK3-20* (91.5)	*JK1* (97.2)
GCB14	PA26	IgG1 (κ)	32.9	4.3E+05	2.0E−03	4.5E−09	*VH4-59* (90.9)	*D2-2*	*JH5* (88.2)	*VK3-15* (98.2)	*JK2* (94.7)
GCB41	PA26	IgG1 (κ)	605.3	8.6E+05	4.9E−04	6.2E−10	*VH1-18* (96.1)	*D6-13*	*JH4* (85.4)	*VK1-16* (100)	*JK3* (92.1)
GCB53	PA26	IgG1 (κ)	222.6	1.9E+06	4.0E−04	2.5E−10	*VH3-73* (95.9)	*D3-3*	*JH6* (83.9)	*VK1D-39* (89.5)	*JK1* (94.7)
GCB59	PA26	IgG1 (λ)	307.6	1.7E+06	1.2E−03	6.8E−10	*VH3-21* (86.8)	*D2-15*	*JH6* (77.4)	*VL3-21* (92.1)	*JL2* (91.9)
GCC9	PA40	IgG1 (κ)	43.2	1.2E+06	9.6E−04	9.4E−10	*VH4-30-2* (87.6)	*D3-10*	*JH5* (92.2)	*VK1-NL1* (97.5)	*JK4* (97.4)
GCC11	PA40	IgG1 (κ)	55.4	6.3E+05	1.8E−03	2.7E−09	*VH4-39* (95)	*D6-13*	*JH1* (78.3)	*VK3-20* (97.5)	*JK1* (94.7)
GCC13	PA40	IgG1 (κ)	16.1	1.0E+06	7.5E−04	9.8E−10	*VH3-21* (96.2)	*D5-24*	*JH2* (88.7)	*VK4-1* (97.6)	*JK1* (100)
GCC21	PA40	IgG1 (κ)	68.1	4.5E+05	2.1E−04	1.1E−09	*VH3-23* (95.4)	*D5-24*	*JH4* (89.6)	*VK3-11* (97.1)	*JK4* (97.3)
GCD10	PA96	IgG3 (λ)	241.7	2.0E+06	3.3E−03	1.9E−09	*VH3-30* (85.1)	*D6-25*	*JH3* (82)	*VK4-1* (89.9)	*JK4* (86.8)
GCD22	PA96	IgG1 (κ)	205.4	4.1E+05	1.9E−03	5.1E−09	*VH3-11* (87.2)	*D2-2*	*JH5* (86.3)	*VK3-11* (93.6)	*JK5* (100)
GCD27	PA96	IgG1 (κ)	166.4	9.9E+05	1.5E−04	1.5E−10	*VH7-4-1* (89.2)	*D3-10*	*JH6* (79.4)	*VK1-27* (89.6)	*JK3* (94.7)
GCE402	PA65	IgG1 (κ)	107.8	1.2E+06	4.5E−04	4.0E−10	*VH4-4* (84.9)	*D6-13*	*JH6* (85.5)	*VK1D-39* (85.5)	*JK3* (92.1)
GCE536	PA65	IgG1 (κ)	61.4	6.6E+05	1.1E−04	1.8E−10	*VH1-46 (87.9)*	*D2-2*	*JH6 (85.5)*	*VK3-20 (91.5)*	*JK2 (92.1)*
Clone 3092		mIgG1 (κ)	61.0	5.9E+05	3.4E−04	5.7E−10					
Clone 1089		mIgG1 (κ)	1080.0	1.8E+05	7.9E−05	4.4E−10					
MOR103		IgG1 (λ)	90.0	2.7E+05	1.5E−05	1.9E−10					
Namilumab		IgG1 (κ)	75.3	3.1E+05	7.7E−05	2.4E−10					

ELISA, enzyme-linked immunosorbent assay; GL, germline; GM-CSF, granulocyte–macrophage colony-stimulating facto; mAb, monoclonal antibody; SPR, surface plasmon resonance.

Binding properties to GM-CSF (EC50, as determined using ELISA, and *K*_a_, *K*_d_ and *K*_D_, as determined by SPR) and V(D)J gene usage of heavy chain and light chain are reported. Binding properties of four control mAbs (mouse clones 3092 and 1089 and therapeutic anti-GM-CSF antibodies MOR103 and Namilumab) are reported at the bottom of the table. None of the autoantibodies isolated cross-reacted with mouse or rat GM-CSF.

## References

[b1] Watanabe M., Uchida K., Nakagaki K., Trapnell B. C. & Nakata K. High avidity cytokine autoantibodies in health and disease: pathogenesis and mechanisms. Cytokine Growth Factor Rev. 21, 263–273 (2010). 20417147 10.1016/j.cytogfr.2010.03.003

[b2] Puel A. *et al.* Autoantibodies against IL-17A, IL-17F, and IL-22 in patients with chronic mucocutaneous candidiasis and autoimmune polyendocrine syndrome type I. J. Exp. Med. 207, 291–297 (2010). 20123958 10.1084/jem.20091983 PMC2822614

[b3] Madariaga L. *et al.* Detection of anti-interferon-gamma autoantibodies in subjects infected by *Mycobacterium tuberculosis*. Int. J. Tuberc. Lung Dis. 2, 62–68 (1998). 9562113

[b4] Patel S. Y. *et al.* Anti-IFN-gamma autoantibodies in disseminated nontuberculous mycobacterial infections. J. Immunol. 175, 4769–4776 (2005). 16177125 10.4049/jimmunol.175.7.4769

[b5] Trapnell B. C., Carey B. C., Uchida K. & Suzuki T. Pulmonary alveolar proteinosis, a primary immunodeficiency of impaired GM-CSF stimulation of macrophages. Curr. Opin. Immunol. 21, 514–521 (2009). 19796925 10.1016/j.coi.2009.09.004 PMC2779868

[b6] Casadevall N. *et al.* Pure red-cell aplasia and antierythropoietin antibodies in patients treated with recombinant erythropoietin. New Engl. J. Med. 346, 469–475 (2002). 11844847 10.1056/NEJMoa011931

[b7] Sergeeva A., Ono Y., Rios R. & Molldrem J. J. High titer autoantibodies to GM-CSF in patients with AML, CML and MDS are associated with active disease. Leukemia 22, 783–790 (2008). 18216869 10.1038/sj.leu.2405104 PMC3403381

[b8] Sethu S. *et al.* Immunoglobulin G1 and immunoglobulin G4 antibodies in multiple sclerosis patients treated with IFNbeta interact with the endogenous cytokine and activate complement. Clin. Immunol. 148, 177–185 (2013). 23770627 10.1016/j.clim.2013.05.008 PMC3779799

[b9] Pohl M. A., Rivera J., Nakouzi A., Chow S. K. & Casadevall A. Combinations of monoclonal antibodies to anthrax toxin manifest new properties in neutralization assays. Infect. Immun. 81, 1880–1888 (2013). 23509144 10.1128/IAI.01328-12 PMC3676002

[b10] Chen C. *et al.* Potent neutralization of botulinum neurotoxin/B by synergistic action of antibodies recognizing protein and ganglioside receptor binding domain. PLoS ONE 7, e43845 (2012). 22952786 10.1371/journal.pone.0043845 PMC3430616

[b11] Nowakowski A. *et al.* Potent neutralization of botulinum neurotoxin by recombinant oligoclonal antibody. Proc. Natl Acad. Sci. USA 99, 11346–11350 (2002). 12177434 10.1073/pnas.172229899 PMC123259

[b12] Montero-Julian F. A., Gautherot E., Wijdenes J., Klein B. & Brailly H. Pharmacokinetics of interleukin-6 during therapy with anti-interleukin-6 monoclonal antibodies: enhanced clearance of interleukin-6 by a combination of three anti-interleukin-6 antibodies. J. Interferon Res. 14, 301–302 (1994). 7861038 10.1089/jir.1994.14.301

[b13] Boyman O., Kovar M., Rubinstein M. P., Surh C. D. & Sprent J. Selective stimulation of T cell subsets with antibody-cytokine immune complexes. Science 311, 1924–1927 (2006). 16484453 10.1126/science.1122927

[b14] Shibata Y. *et al.* GM-CSF regulates alveolar macrophage differentiation and innate immunity in the lung through PU.1. Immunity 15, 557–567 (2001). 11672538 10.1016/s1074-7613(01)00218-7

[b15] Uchida K. *et al.* GM-CSF autoantibodies and neutrophil dysfunction in pulmonary alveolar proteinosis. New Engl. J. Med. 356, 567–579 (2007). 17287477 10.1056/NEJMoa062505

[b16] Sakagami T. *et al.* Human GM-CSF autoantibodies and reproduction of pulmonary alveolar proteinosis. New Engl. J. Med. 361, 2679–2681 (2009). 10.1056/NEJMc0904077 PMC4174270 20042763

[b17] Uchida K. *et al.* High-affinity autoantibodies specifically eliminate granulocyte-macrophage colony-stimulating factor activity in the lungs of patients with idiopathic pulmonary alveolar proteinosis. Blood 103, 1089–1098 (2004). 14512323 10.1182/blood-2003-05-1565

[b18] Wang Y. *et al.* Characterization of pathogenic human monoclonal autoantibodies against GM-CSF. Proc. Natl Acad. Sci. USA 110, 7832–7837 (2013). 23620516 10.1073/pnas.1216011110 PMC3651501

[b19] Traggiai E. *et al.* An efficient method to make human monoclonal antibodies from memory B cells: potent neutralization of SARS coronavirus. Nat. Med. 10, 871–875 (2004). 15247913 10.1038/nm1080 PMC7095806

[b20] Lefranc M. P. *et al.* IMGT, the international ImMunoGeneTics information system. Nucleic Acids Res. 37, D1006–D1012 (2009). 18978023 10.1093/nar/gkn838 PMC2686541

[b21] Uchida K. *et al.* Granulocyte/macrophage-colony-stimulating factor autoantibodies and myeloid cell immune functions in healthy subjects. Blood 113, 2547–2556 (2009). 19282464 10.1182/blood-2009-05-155689 PMC2656275

[b22] Krinner E. M. *et al.* A human monoclonal IgG1 potently neutralizing the pro-inflammatory cytokine GM-CSF. Mol. Immunol. 44, 916–925 (2007). 16697465 10.1016/j.molimm.2006.03.020

[b23] Steidl S., Ratsch O., Brocks B., Durr M. & Thomassen-Wolf E. *In vitro* affinity maturation of human GM-CSF antibodies by targeted CDR-diversification. Mol. Immunol. 46, 135–144 (2008). 18722015 10.1016/j.molimm.2008.07.013

[b24] Niu L., Heaney M. L., Vera J. C. & Golde D. W. High-affinity binding to the GM-CSF receptor requires intact N-glycosylation sites in the extracellular domain of the beta subunit. Blood 95, 3357–3362 (2000). 10828016

[b25] Hansen G. *et al.* The structure of the GM-CSF receptor complex reveals a distinct mode of cytokine receptor activation. Cell 134, 496–507 (2008). 18692472 10.1016/j.cell.2008.05.053

[b26] Behrens F. *et al.* MOR103, a human monoclonal antibody to granulocyte-macrophage colony-stimulating factor, in the treatment of patients with moderate rheumatoid arthritis: results of a phase Ib/IIa randomised, double-blind, placebo-controlled, dose-escalation trial. Ann. Rheum. Dis. 74, 1058–1064 (2014). 24534756 10.1136/annrheumdis-2013-204816 PMC4431325

[b27] Respaud R., Vecellio L., Diot P. & Heuze-Vourc’h N. Nebulization as a delivery method for mAbs in respiratory diseases. Expert Opin. Drug Deliv. 12, 1027–1039 (2015). 25557066 10.1517/17425247.2015.999039

[b28] Tabrizi M., Bornstein G. G. & Suria H. Biodistribution mechanisms of therapeutic monoclonal antibodies in health and disease. AAPS J. 12, 33–43 (2010). 19924542 10.1208/s12248-009-9157-5 PMC2811642

[b29] Di Zenzo G. *et al.* Pemphigus autoantibodies generated through somatic mutations target the desmoglein-3 cis-interface. J. Clin. Invest. 122, 3781–3790 (2012). 22996451 10.1172/JCI64413 PMC3461925

[b30] Mietzner B. *et al.* Autoreactive IgG memory antibodies in patients with systemic lupus erythematosus arise from nonreactive and polyreactive precursors. Proc. Natl Acad. Sci. USA 105, 9727–9732 (2008). 18621685 10.1073/pnas.0803644105 PMC2474524

[b31] Schroeder K., Herrmann M. & Winkler T. H. The role of somatic hypermutation in the generation of pathogenic antibodies in SLE. Autoimmunity 46, 121–127 (2013). 23181829 10.3109/08916934.2012.748751

[b32] Corti D. *et al.* A neutralizing antibody selected from plasma cells that binds to group 1 and group 2 influenza A hemagglutinins. Science 333, 850–856 (2011). 21798894 10.1126/science.1205669

[b33] Corti D. *et al.* Cross-neutralization of four paramyxoviruses by a human monoclonal antibody. Nature 501, 439–443 (2013). 23955151 10.1038/nature12442

[b34] Pappas L. *et al.* Rapid development of broadly influenza neutralizing antibodies through redundant mutations. Nature 516, 418–422 (2014). 25296253 10.1038/nature13764

[b35] Lin R. H., Mamula M. J., Hardin J. A. & Janeway C. A. Jr. Induction of autoreactive B cells allows priming of autoreactive T cells. J. Exp. Med. 173, 1433–1439 (1991). 1851798 10.1084/jem.173.6.1433 PMC2190847

[b36] Dabritz J. *et al.* Granulocyte macrophage colony-stimulating factor auto-antibodies and disease relapse in inflammatory bowel disease. Am. J. Gastroenterol. 108, 1901–1910 (2013). 24145675 10.1038/ajg.2013.360

[b37] Kitamura T. *et al.* Serological diagnosis of idiopathic pulmonary alveolar proteinosis. Am. J. Respir. Crit. Care Med. 162, 658–662 (2000). 10934102 10.1164/ajrccm.162.2.9910032

[b38] Inoue Y. *et al.* Characteristics of a large cohort of patients with autoimmune pulmonary alveolar proteinosis in Japan. Am. J. Respir. Crit. Care Med. 177, 752–762 (2008). 18202348 10.1164/rccm.200708-1271OC PMC2720118

