# Supplementary material for: Neutralization and clearance of GM-CSF by autoantibodies in pulmonary alveolar proteinosis
Source: Nat Commun. 2015 Jun 16;6:7375. doi: 10.1038/ncomms8375 (PMC4477037; doi:10.1038/ncomms8375)
Supplement: Supplementary Information — Supplementary Figures 1-3, Supplementary Tables 1-2 [file ncomms8375-s1.pdf]

SUPPLEMENTARY INFORMATION

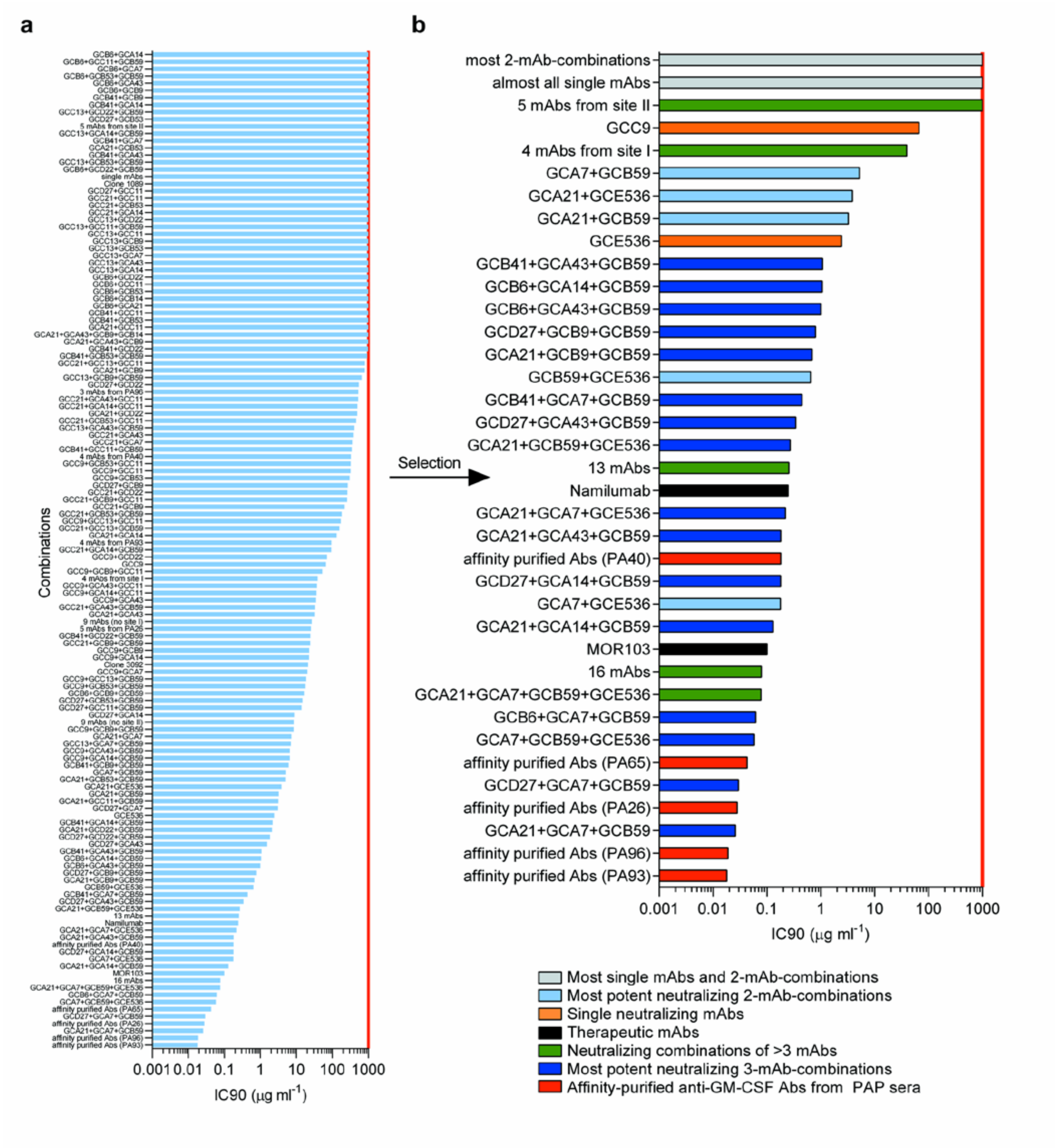

Supplementary Figure 1. *In vitro* neutralization of GM-CSF by single or multiple antibodies. (**a**) Rank of neutralizing activity (IC90 values) of 140 different combinations of GM-CSF mAbs. (**b**) A selection of the most potent neutralizing combinations is shown and compared to therapeutic anti-GM-CSF antibodies (MOR103 and Namilumab) and to autoantibodies purified from PAP sera.

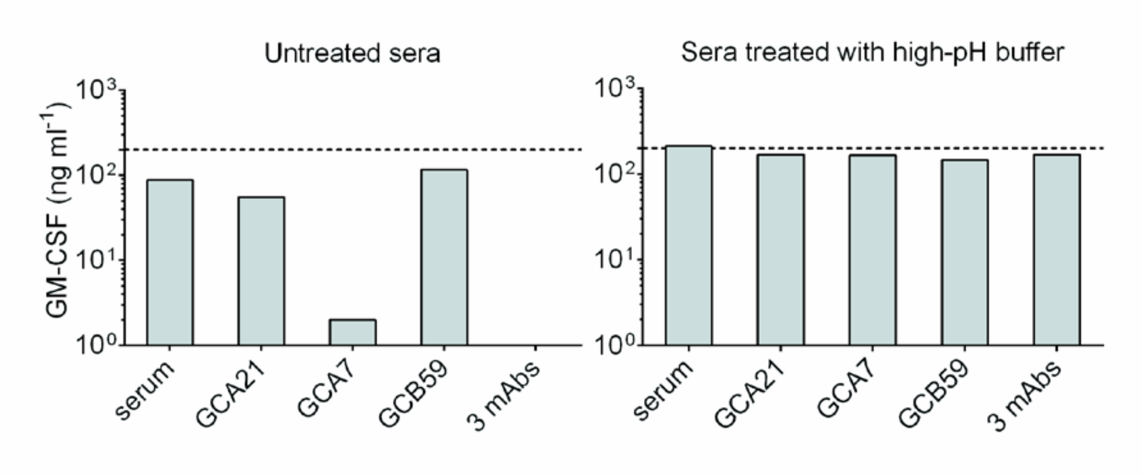

Supplementary Figure 2. A sandwich ELISA to detect GM-CSF in the presence of specific antibodies. A fixed amount of GM-CSF was added to mouse serum together with three monoclonal antibodies (GCA21, GCA7, GCB59) added separately or in combination. The quantification of GM-CSF was performed by a sandwich ELISA using an antibody specific for site II for capture and site I for detection. Serial dilutions of serum in neutral (left) or alkaline buffer (right) were added and GM-CSF concentration was determined with reference to a GM-CSF standard. The dotted line represents the concentration of GM-CSF measured in the absence of antibodies.

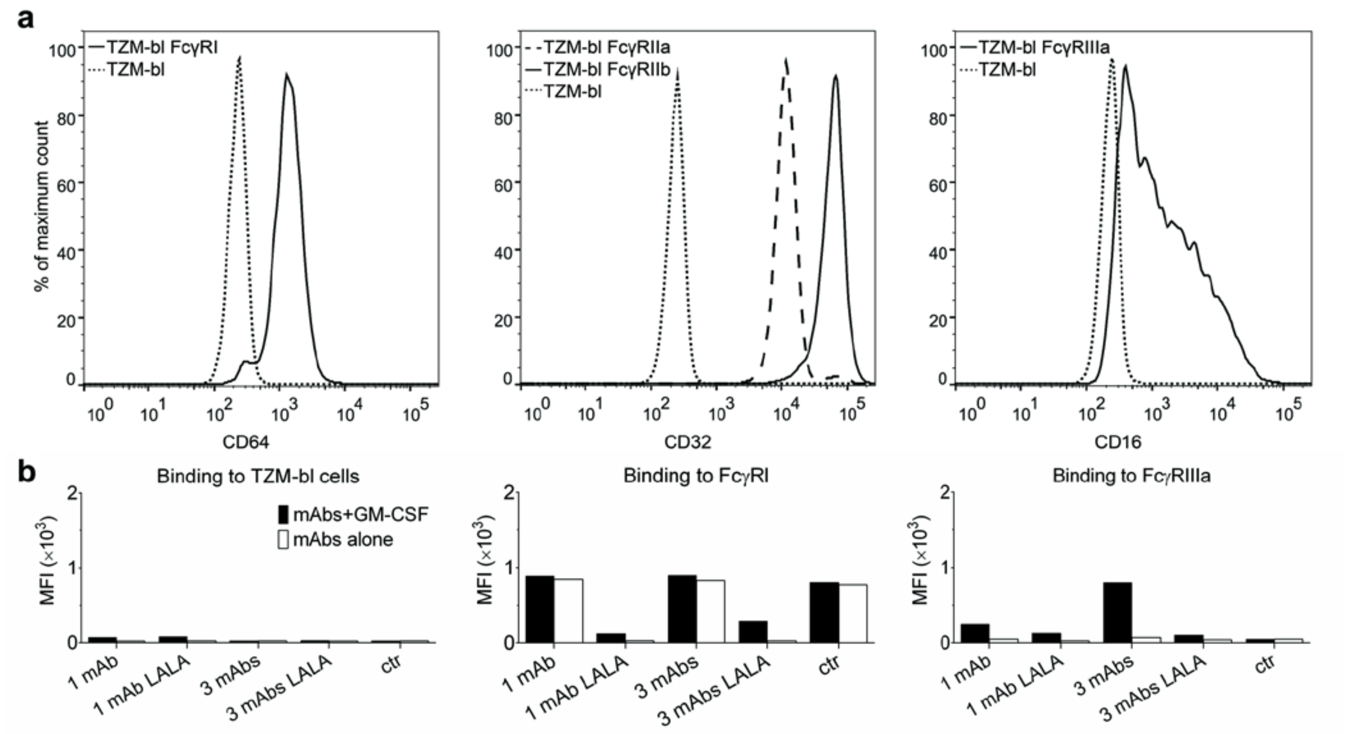

Supplementary Figure 3. Binding of immune complexes to TZM-bl cells expressing different FcγRs. (**a**) Expression of FcγRs on TZM-bl cells transfected with FcγRI, FcγRIIa/b and FcγRIIIa, as detected by antibodies to CD64, CD32 and CD16, respectively. Untransfected TZM-bl cells were used as negative control. (**b**) Shown is also the binding of GM-CSF immune complexes containing one or three antibodies to TZM-bl cells expressing FcγRI or FcγRIIIa or to untransfected TZM-bl cells.

Supplementary Table 1

Features of 5 PAP patients

ID	Code	Gender	Birth year	Diagnosis year	Therapy	Follow up (years)	DSS	Serum GM-CSF abs (μg ml^{-1})
PA93	GCA	M	1943	2010	-	2	3	804.5
PA26	GCB	F	1966	2005	-	7	3	454.9
PA40	GCC	F	1954	2005	WLL	7	1	100.6
PA96	GCD	M	1980	2010	-	2	1	741.2
PA65	GCE	F	1959	2005	WLL	7	1	46.0

WLL = whole lung lavage; DSS = Disease Severity Score calculated at the time of sample collection; abs = antibodies

Supplementary Table 2
Somatic mutations in the V segment critically contribute to the specificity of GM-CSF autoantibodies

mAb	Version	EC50 (ng ml^{-1})	Ka (M s^{-1})	Kd (s^{-1})	KD (M)
**GCA21**	*WT*	33.3	1.3E+06	5.5E-04	5.7E-10
	VK UCA	77.3	3.1E+06	2.6E-02	1.1E-08
	VH UCA	nc	nc	nc	nc
	UCA	nc	nc	nc	nc
	UCA - HCDR3 WT	nc	nc	nc	nc
**GCA7**	*WT*	52.4	1.8E+05	3.1E-05	2.3E-10
	VK UCA	64.8	4.9E+04	1.7E-05	3.5E-10
	VH UCA	nc	nc	nc	nc
	UCA	7724	nc	nc	nc
	UCA - HCDR3 WT	354.2	3.4E+04	2.1E-03	6.3E-08
**GCB59**	*WT*	428.7	1.0E+06	1.7E-03	1.8E-09
	VL UCA	24039	1.3E+06	1.0E-02	8.4E-09
	VH UCA	nc	nc	nc	nc
	UCA	nc	nc	nc	nc
	UCA - HCDR3 WT	nc	nc	nc	nc
**GCB9**	*WT*	322	3.1E+05	7.5E-04	2.5E-09
	VK UCA	258.3	4.8E+05	2.8E-03	6.2E-09
	VH UCA	956	4.2E+05	3.9E-03	1.5E-08
	UCA	6765	1.7E+05	1.2E-02	8.2E-08
	UCA - HCDR3 WT	3664	5.9E+04	8.5E-03	1.5E-07
**GCA43**	*WT*	258	2.5E+05	1.5E-04	6.2E-10
	VK UCA	286.7	3.0E+05	1.7E-04	6.5E-10
	VH UCA	nc	nc	nc	nc
	UCA	nc	nc	nc	nc
	UCA - HCDR3 WT	nc	nc	nc	nc
**GCB6**	*WT*	31.1	5.2E+05	4.4E-04	1.0E-09
	VK UCA	35.1	6.5E+05	1.0E-02	1.9E-08
	VH UCA	nc	nc	nc	nc
	UCA	nc	nc	nc	nc
	UCA - HCDR3 WT	nc	nc	nc	nc
**GCE536**	*WT*	54.3	5.8E+05	1.4E-04	2.5E-10
	VK UCA	37.5	7.3E+05	9.5E-04	1.3E-09
	VH UCA	56.3	4.7E+04	5.4E-05	1.1E-09
	UCA	19773	2.4E+03	8.7E-05	9.0E-08
	UCA - HCDR3 WT	13.7	5.5E+05	6.0E-03	1.0E-08

Binding of WT, UCA, UCA-HCDR3 WT and shuffled variants VK/VL UCA and VH UCA to GM-CSF as measured by ELISA and SPR. nc = not calculable
